# Prospective control of steering through multiple waypoints

**DOI:** 10.1167/jov.24.8.1

**Published:** 2024-08-01

**Authors:** A. J. Jansen, Brett R. Fajen

**Affiliations:** 1 Cognitive Science Department, Rensselaer Polytechnic Institute, Troy, NY, USA

**Keywords:** steering, visual control, gaze behavior, optic flow, drones

## Abstract

Some locomotor tasks involve steering at high speeds through multiple waypoints within cluttered environments. Although in principle actors could treat each individual waypoint in isolation, skillful performance would seem to require them to adapt their trajectory to the most immediate waypoint in anticipation of subsequent waypoints. To date, there have been few studies of such behavior, and the evidence that does exist is inconclusive about whether steering is affected by multiple future waypoints. The present study was designed to address the need for a clearer understanding of how humans adapt their steering movements in anticipation of future goals. Subjects performed a simulated drone flying task in a forest-like virtual environment that was presented on a monitor while their eye movements were tracked. They were instructed to steer through a series of gates while the distance at which gates first became visible (i.e., lookahead distance) was manipulated between trials. When gates became visible at least 1-1/2 segments in advance, subjects successfully flew through a high percentage of gates, rarely collided with obstacles, and maintained a consistent speed. They also approached the most immediate gate in a way that depended on the angular position of the subsequent gate. However, when the lookahead distance was less than 1-1/2 segments, subjects followed longer paths and flew at slower, more variable speeds. The findings demonstrate that the control of steering through multiple waypoints does indeed depend on information from beyond the most immediate waypoint. Discussion focuses on the possible control strategies for steering through multiple waypoints.

## Introduction

### Steering through multiple waypoints

Many locomotor tasks involve steering through cluttered environments at moderate to high speeds, moving smoothly from one waypoint to the next while in some cases avoiding obstacles. In humans, such behavior is a key component of skilled actions such as slalom skiing, glade skiing, and mountain biking (Decroix et al., 2017; Schläppi, Urfer, & Kredel, 2016; Wilkie, Wann, & Allison, 2008). Flying animals, including birds and flying insects, also exhibit impressive abilities to steer through a series of openings within clutter (Antolin & Taylor, 2023; Baird & Dacke, 2016; Brighton et al., 2023; Fabian, Sumner, Wardill, & Gonzalez-Bellido, 2022; Henningsson, 2021; Khandelwal & Hedrick, 2020; Lin, Ros, & Biewener, 2014; Ravi et al., 2022). Parallel work in the field of robotics focuses on algorithms for guiding ground vehicles and aerial robots along smooth trajectories through multiple waypoints (Barry, Florence, & Tedrake, 2018; Foehn, Romero, & Scaramuzza, 2021; Kaufmann et al., 2023).

Up to this point, the majority of theoretical and empirical work on the control of steering in humans has focused on steering to a single target (Fajen, 2001; Li & Cheng, 2013; Wann & Wilkie, 2004; Wilkie & Wann, 2003) or along a winding road (Land & Horwood, 1995; Land & Lee, 1994; Lappi & Mole, 2018; Salvucci & Gray, 2004; Wallis, Chatziastros, & Bülthoff, 2002). Much less is known about the control strategies used to steer through multiple waypoints. In generalizing from single-target to multiple-waypoint tasks, a critical open question (and the focus of the present study) concerns the role of information from waypoints that lie beyond the most immediate one. Under some conditions, steering may be guided entirely by information from the most immediate waypoint, with information from future waypoints playing a negligible role. Behavior could then be well captured by single-target control strategies, with the target reset to the next waypoint as each waypoint is acquired. However, unless waypoints are spaced far apart in depth and positioned along a smooth, predictable path (e.g., Tuhkanen et al., 2019; Tuhkanen, Pekkanen, Wilkie, & Lappi, 2021; Wilkie et al., 2008), focusing entirely on the most immediate waypoint and neglecting what lies beyond could negatively affect performance. Observers may be forced to make abrupt velocity changes, follow less efficient trajectories, or miss waypoints altogether. By using a control strategy that adapts how one approaches the most immediate waypoint in anticipation of future waypoints, observers may be able to maintain performance under more challenging conditions.

The aim of the present study was to determine whether humans do in fact prospectively control steering with respect to waypoints beyond the most immediate one and, if so, which properties of future waypoints (e.g., position, orientation) affect their trajectory. Such data are essential to formulating plausible strategies for the prospective control of steering through multiple waypoints. Unfortunately, the existing literature provides at best an incomplete picture of the role of future-waypoint information. In one of the earlier studies of steering behavior, Land and Horwood (1995) had subjects drive along a winding road in a simulator based on information from 1° horizontal slices of the visual field. Although steering along a winding road differs from steering through multiple waypoints, this study revealed some useful insights into the relative contributions of information from near and far regions of the visual field by manipulating the vertical position of the horizontal slices. When far-road information was occluded, subjects were able to maintain lane position, but steering behavior became less stable. This led Land and Horwood to suggest that information from more distant regions of the visual field allows drivers to anticipate upcoming trajectory constraints and generate smoother, more stable trajectories (see also Lehtonen, Lappi, Koirikivi, & Summala, 2014; Salvucci & Gray, 2004).

One of the few studies on steering through multiple waypoints is that by Wilkie et al. (2008), who had subjects perform a slalom steering task in a simulated environment, steering a stationary bike through a series of gates that were positioned at the apexes of a sum-of-sines path. One of the key findings from this study was that subjects often tracked the upcoming gate (Gate*_N_*) with their gaze until the gate reached a certain distance, at which time gaze shifted to the next gate (Gate*_N_*_+1_). A similar pattern of eye movements has been observed in other studies of driving along a winding road (Lehtonen, Lappi, Kotkanen, & Summala, 2013; Lehtonen et al., 2014). A plausible explanation for such behavior is that, by shifting gaze to Gate*_N_*_+1_ before reaching Gate*_N_*, subjects were better able to detect the information needed to anticipate future waypoints, which in turn might allow for smoother steering behavior. However, the empirical support for this hypothesis in the follow-up experiments conducted by Wilkie et al. (2008) is inconclusive.

In Experiment 2 of Wilkie et al. (2008), only one gate was visible at a time and subjects could manually adjust the “switch time” (i.e., the time before reaching the upcoming gate at which that gate disappeared and the next one appeared) to match their preferences. Although subjects who preferred earlier switch times tended to exhibit smoother steering (i.e., lower angular acceleration) and vice versa, the correlation between switch time and angular acceleration was weak (*r* = −0.13). Switch time was manipulated by the experimenters in Experiment 3 with similar results: Steering was least smooth (i.e., angular acceleration was greatest) in the late switch time conditions but the effect was only marginally significant (*p* = 0.056).

One might infer from the weak effects of switch time on steering smoothness that information from beyond the upcoming gate plays a negligible role. However, the conditions that were used in Wilkie et al. (2008) were not ideally suited to test the potential role of such information because successive gates were spaced far apart (∼20 to ∼40 m). With speed fixed at 8 m/s, subjects had 2-1/2 to 5 seconds between gates to adjust steering, which is plenty of time to generate a smooth trajectory even in the most extreme late switch time condition. This could explain why the effect of switch time on steering smoothness was not more pronounced.

More recently, Powell, Marshall, Diaz, and Fajen (2024) studied the steering and gaze behavior of skilled drone pilots in a forest-like virtual environment with hoops that were positioned at irregular intervals along a winding path. Subjects flew at a faster speed (10–15 m/s) than in Wilkie et al. (2008) even though hoops varied in relative orientation and were positioned closer together; the average distance between hoops was 17.6 m. Under such conditions, the benefit of using information from future hoops is potentially greater than in Wilkie et al. (2008). Indeed, the angle at which the drone approached each upcoming hoop varied systematically with the position of the subsequent hoop. This is consistent with a control strategy that relies on information from the two upcoming waypoints (*N* and *N*+1). However, the evidence is somewhat indirect in that it was based on a significant correlation between the angular position of Hoop *N*+1 and the quadcopter's approach angle to Hoop *N*. In addition, subjects in that study were skilled drone pilots with many hours of experience steering through cluttered environments at high speeds. This leaves open the question of whether the use of future-waypoint information is something that only emerges with skill after extensive practice or is a more general characteristic of steering behavior.

### The present study

The present study builds upon Powell et al. (2024) by more directly testing the role of information from beyond the upcoming waypoint and by testing subjects who were not experienced first-person view (FPV) drone pilots. As in Powell et al., the task involved steering a quadcopter through a forest-like virtual environment. Subjects were instructed to steer through a series of rectangular gates that were placed at various orientations along an irregularly shaped, closed-loop path. To determine how performance depended on information from beyond the most immediate gate, we manipulated how far in advance gates became visible (i.e., lookahead distance). This is similar to the manipulation used in Wilkie et al. (2008) but differs in that, when a gate appeared, it remained visible until the observer passed through it. We also tracked subjects’ eye movements to better understand how steering performance was related to gaze shifts between the most immediate gate and future gates. Finally, we aimed to determine whether the role of information about future waypoints depends on how irregularly they are distributed; in particular, we tested whether such information played a more important role when waypoints were less evenly spaced. As such, the experiment included two conditions that differed in terms of the configuration of gates.

## Methods

### Participants

Sixteen naïve subjects (11 men, five women) participated in this study, recruited from the undergraduate student body at Rensselaer Polytechnic Institute. Ages ranged from 18 to 24 years old, with a mean age of 20.8 years. All subjects had normal or corrected-to-normal vision, and none had any impairments that impacted their ability to use a game controller. Fifteen subjects were right-handed and one left-handed. Seven participants reported playing any video games for over 5 hours a week; one between 2 and 5 hours; four under 2 hours; and four never or rarely played video games. When asked about FPV games navigating a three-dimensional environment, five reported playing such games more than 5 hours a week; one between 2 and 5 hours; three under 2 hours; and seven never or rarely. The protocol (#2069) was approved by the Rensselaer Polytechnic Institute Institutional Review Board. All subjects gave their written informed consent before participation. One subject received course credit for participation and 15 received monetary compensation.

### Hardware

**Figure 1.**
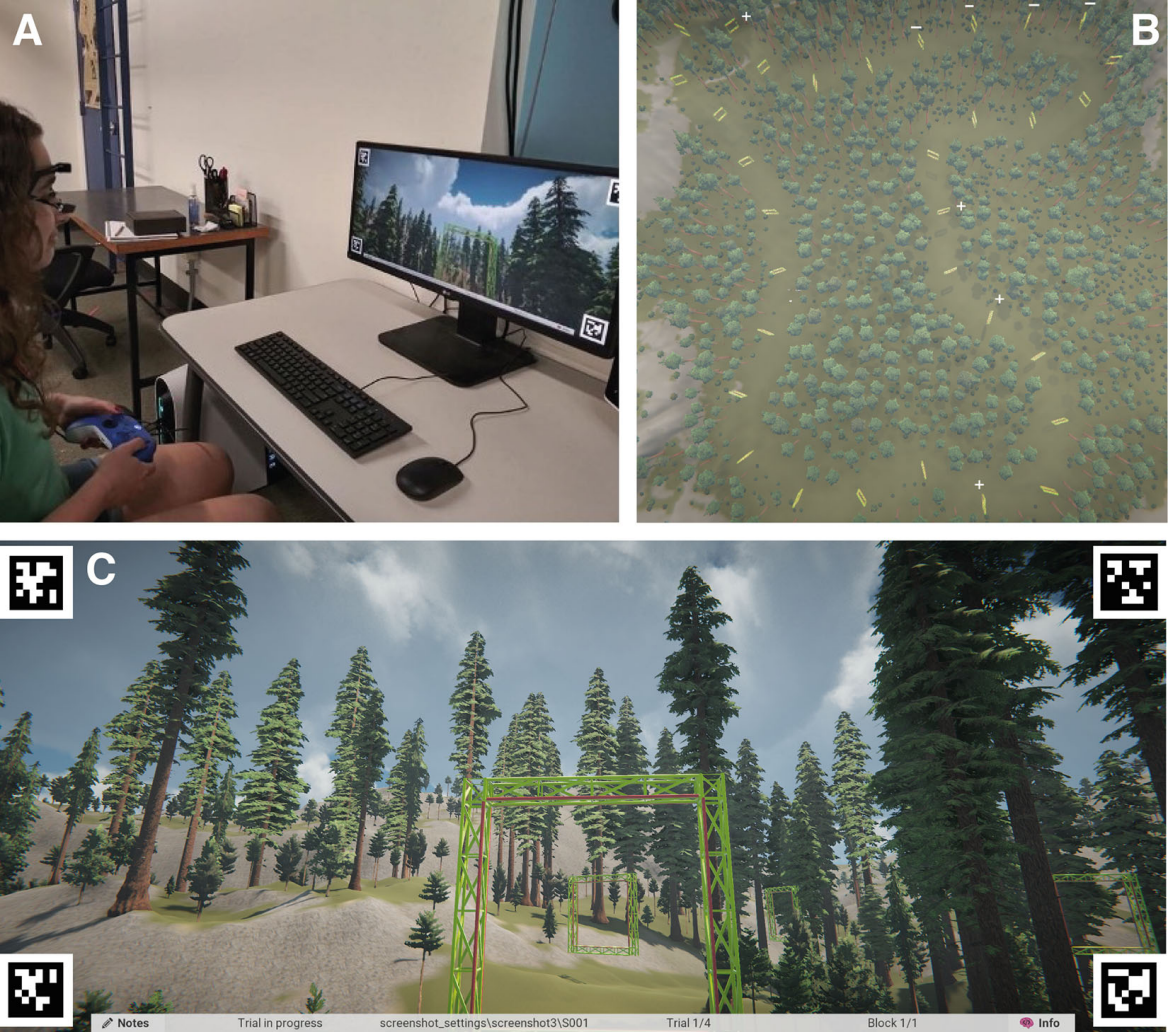
(**A**) Experimental setup. (**B**) Bird's-eye view of virtual environment showing the path and yellow gates. White + and – signs denote gates for which *N*+1 fixations tended to occur later (positive) or earlier (negative) (see Gaze Behavior section of Results for further explanation). (**C**) First-person view of the scene.

The virtual environment was generated in Unity 2019.4.29f1 running on an Alienware Aurora R12 (Dell, Round Rock, TX) equipped with an NVIDIA GeForce RTX 3090 graphics card (NVIDIA, Santa Clara, CA) and 3.50-GHz 11th Gen Intel Core i9-11900KF processor (Intel, Santa Clara, CA). The environment was displayed on a 69 cm × 30 cm LG UltraWide monitor (LG Electronics, Seoul, South Korea) at 2560 × 1080 resolution with a 60-Hz refresh rate (see Figure 1A). Subjects were seated approximately 1 meter away from the monitor, which subtended a horizontal visual angle of 38° and a vertical angle of 17° when viewed from that distance. An Xbox Series X Wireless Controller (Microsoft, Redmond, WA) was used to control movement of the drone.

### Virtual environment

The virtual environment was created in Unity using GaiaPro, an asset from the Unity Asset Store (author Procedural Worlds). The terrain was largely grass textured with small variations in elevation and occasional patches of stone. Trees were placed with medium density throughout the environment, leaving a clear track along which 32 gates were placed at intervals of approximately 23 meters. The track formed an irregularly shaped loop approximately 740 meters long and was marked only by the absence of trees (i.e., there was no path or trail) (see Figures 1B and 1C). The session framework utilized the Unity Experiment Framework (UXF) package to initialize experiment settings and for trial management and data logging (Brookes, Warburton, Alghadier, Mon-Williams, & Mushtaq, 2020).

The simulated drone and drone controller were assets purchased from the Unity Asset Store (Drones Bundle Package and FPV Drone controller; author Mario Haberle). The drone had a simulated mass of 0.3 kg and maximum speed of 12.5 m/s, and the drone camera had a field of view of 70°. The rotation (yaw) rate of the drone was controlled by the *x*-axis of the left joystick and forward/backward thrust was controlled by the *y*-axis of the right joystick. The altitude of the drone relative to a fixed reference level in the simulated environment was constant.

Although the control mapping was simplified to accommodate novices, the experience for subjects more closely resembled that of flying than driving for two reasons. First, because the altitude of the drone was constant relative to a fixed reference level, its height above the ground changed with variations in terrain height, unlike in a car. Second, because subjects had independent control of rotation rate and forward/backward thrust, they could adjust turning rate without changing speed and even rotate the drone without translating. Supplementary Movie S1 is a recording of a segment of an experimental trial and illustrates the experience for subjects.

### Design and procedure

Subjects were briefed on the procedure of the study, and written consent was obtained. Participants were then fitted with the eye tracker, after which they practiced flying around freely in a practice virtual environment that was similar but not identical to the test environment. The practice session lasted for at least 5 minutes and allowed subjects to become familiar with the controller and drone dynamics.

**Figure 2.**
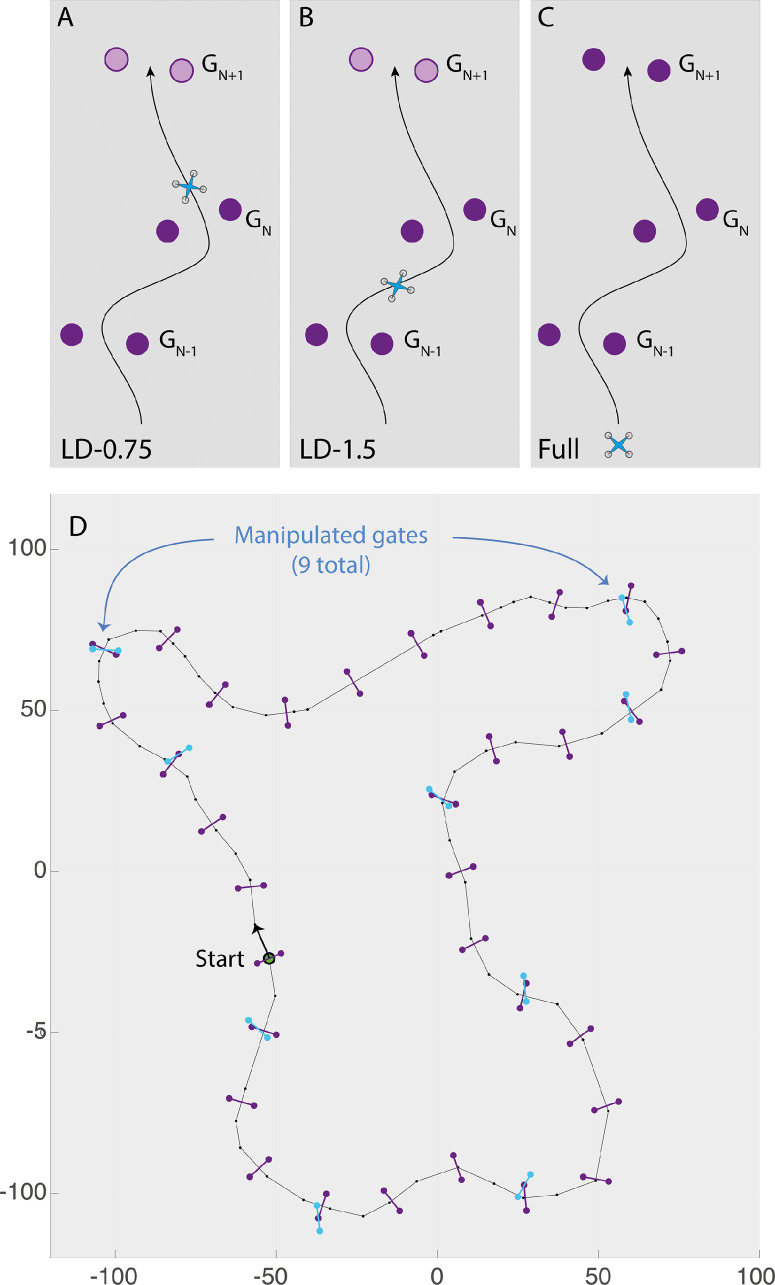
(**A**–**C**) Illustration of the lookahead-distance (LD) manipulation. (**A**) and (**B**) show the position of the quadcopter when Gate*_N_*_+1_ first appeared in the LD-0.75 and LD-1.5 conditions. In the LD-Full condition (**C**), all gates appeared at the beginning of the trial and remained visible. (**D**) Top–down view of path showing position of gates. The purple gates show the positions of the gates in Configuration A. In Configuration B, the positions and orientations of nine of the 32 gates were manipulated (cyan gates), and the remaining 21 gates were in the same positions and orientations as in Configuration A.

The main experiment consisted of 10 blocks, each of which included two laps around the track for a total of 20 laps per session. There were two independent variables: lookahead distance and configuration. Lookahead distance affected how far in advance gates became visible and was fixed within each block but varied across blocks. There were five lookahead distances (LD-0.75, LD-1.0, LD-1.5, LD-2.0, and LD-Full), each measured as a proportion of segments between gates. For example, in the LD-0.75 condition, Gate*_N_*_+1_ did not appear until the distance remaining to that gate was 3/4 of the segment distance between Gate*_N_* and Gate*_N_*_+1_ (see Figure 2A and Supplementary Movie S2). In the LD-1.5 condition, Gate*_N_*_+1_ appeared at the moment that the quadcopter was halfway between Gate*_N_*_–__1_ and Gate*_N_* (see Figure 2B and Supplementary Movie S3). In the LD-Full condition, all 32 gates were added to the scene at the beginning of the trial and remained visible (see Supplementary Movie S1).

The second independent variable was configuration. In Configuration A, gates were perpendicular to and centered on the (non-rendered) path; in Configuration B, nine of the 32 gates were offset from the path, and their orientation was manipulated (see Figure 2D). In each block, one of the 10 (5 lookahead distances × 2 configurations) conditions was selected, with the order of conditions randomized across subjects.

During the main experiment, subjects were instructed to steer through each gate while flying as quickly as was possible without losing control. They were also instructed not to backtrack if they missed a gate. If the drone flew too far off the path, the drone position was reset to the beginning of the lap.

### Eye tracking

Subjects wore a Pupil Labs Core eye tracking device (Pupil Labs, Berlin, Germany), consisting of a glasses-like frame with two eye cameras and a world camera. The eye cameras recorded at 200 Hz and the world camera at 30 Hz. Raw gaze data and accuracy data were captured by the Pupil Capture 3.5.1 software. Eye-tracker calibration was performed at the beginning of every block (approximately every 4 minutes) to account for any possible slipping. The calibration procedure was a built-in function of Pupil Capture, consisting of five fixation targets appearing at intervals on the screen. The average error in gaze estimation was under 1° (*M* = 0.95°, min = 0.3°, max = 3.00°).

### Data analyses

Postprocessing was performed using a combination of Pupil Core software and custom scripts written in R (R Foundation for Statistical Computing, Vienna, Austria) and MATLAB (MathWorks, Natick, MA). Positional data (i.e., location and orientation of the drone and gates relative to the environment) were recorded by Unity and used to calculate additional variables. The change in position of the drone over consecutive frames was calculated and used to estimate the speed and instantaneous heading of the drone as well as the overall path length. The approach angle of the drone relative to the upcoming gate was calculated based on the difference between the normal vector of that gate and the instantaneous drone heading.

Following Wilkie et al. (2008), we used mean absolute angular acceleration as a measure of steering smoothness. Angular acceleration was estimated using the following steps. For each trial, we calculated the heading direction time series based on drone position on successive frames. We then divided the difference in heading direction on successive frames by the difference in time, yielding an estimate of angular speed. The angular speed time series was smoothed using a 10-frame rolling window average. Next, we divided the difference in angular speed on successive frames by the difference in time to estimate angular acceleration and applied the same 10-frame rolling window average. The last step was to calculate the mean absolute angular acceleration.

We also calculated activity of the right and left joysticks, which controlled forward–backward thrust and yaw rate, respectively. Both measures were derived from the joystick position time series, which ranged from −1 to +1, using the following equation:

(1) $$\begin{array}{c} \frac{1}{\#frames-1}\sum_{f=1}^{\#frames-1}\left(\frac{\left|p_{f+1}-p_{f}\right|}{t_{f+1}-t_{f}}\right)\; \end{array}$$

where *f* is the frame number, *p* is joystick position, and *t* is time in seconds. This yields the average absolute change in joystick position per second.

If a lap had to be reset because the drone strayed too far from the path, the data from that lap prior to the reset were removed. In addition, data from segments on which the drone missed or collided with a gate were excluded from the analyses of approach angle.

Raw eye tracking data recorded by Pupil Capture were fed to Pupil Player to remove blinks and low confidence readings (<50%). When necessary, pupil detection was post-calculated for certain trials using the eye camera recordings. The surface mapping plugin was used to transform gaze data from eye camera coordinates to monitor screen coordinates, and gaze data were interpolated with positional data based on the time stamp. Subsequently, the gaze data were smoothed using a seven-frame rolling window average to remove the jitter that resulted from alternating eye sampling.

At every frame, gaze position in screen coordinates was transformed to a world point using a Unity function (ScreenToWorldPoint) in order to recover direction. A ray was then cast from the drone in the gaze direction, and any hit object was recorded. If the hit object was the opening within the frame of a gate, a second ray was cast and a secondary hit also recorded. This allowed for detection of fixations of a more distant gate through the center of a closer gate. Hits on a single gate, either the frame or the center, for at least three sequential frames were recorded as a fixation of that gate. The time stamp at which the drone passed through each gate was used to determine which gate was *N* or *N*+1 in any frame.

## Results

### Basic performance measures

**Figure 3.**
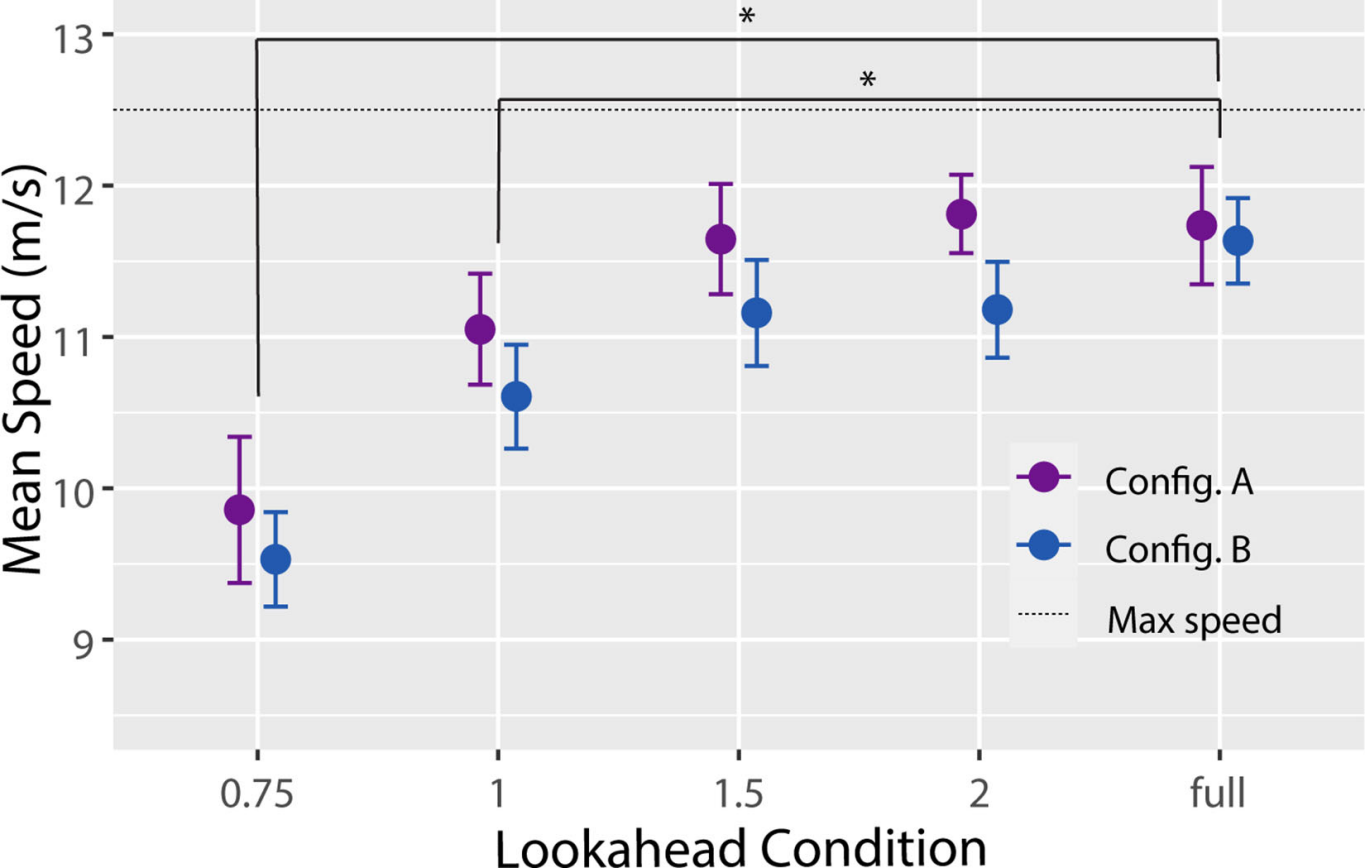
Mean speed as a function of lookahead distance for Configuration A (purple markers) and Configuration B (blue markers). Error bars are 95% confidence intervals with between-subjects variance removed. Asterisks indicate conditions that are significantly different (*p* < 0.05). The dotted line indicates the maximum possible speed.

The first set of analyses focuses on how measures of performance (i.e., average speed, proportion of gates completed, and number of collisions) were affected by lookahead distance and configuration. Average speed varied across subjects and conditions between 9.18 and 12.23 m/s, with an overall average of 10.95 m/s. A two-way repeated-measures analysis of variance (ANOVA) revealed a significant main effect of lookahead distance, *F*(4, 60) = 46.61, *p* < 0.001, η^2^ = 0.63, indicating that subjects maintained slower speeds in the shorter lookahead distance conditions (see Figure 3). Planned comparisons confirmed that average speed was significantly reduced compared with the LD-Full condition in the LD-0.75 and LD-1.0 conditions but not in the LD-1.5 and LD-2.0 conditions. The main effect of configuration was also significant, *F*(1, 15) = 9.65, *p* < 0.01, η^2^ = 0.07, indicating that subjects flew slightly slower in Configuration B, most likely due to the manipulated offset and orientation of nine of the 32 gates in that configuration. The lookahead distance × configuration interaction was not significant, *F*(4, 60) = 0.51, *p* = 0.73, η^2^ = 0.01.

The proportion of gates completed was consistently high across all conditions (*M* = 0.998) and was not significantly affected by lookahead distance, *F*(4, 60) = 1.0, *p* = 0.41, η^2^ = 0.04; configuration, *F*(1, 15) = 1.0, *p* = 0.33, η^2^ = 0.01; or their interaction, *F*(4, 60) = 1.0, *p* = 0.41, η^2^ = 0.04. (The F-ratios are all very close to 1.0 because the proportion of gates completed was 1.0 for most subjects.) Similarly, the number of collisions per lap was low (*M* = 0.944), with no significant effects of lookahead distance, *F*(4, 60) = 1.34, *p* = 0.27, η^2^ = 0.02; configuration, *F*(1, 15) = 1.63, *p* = 0.22, η^2^ = 0.01; or their interaction, *F*(4, 60) = 0.72, *p* = 0.58, η^2^ = 0.03. The mean proportion of gates completed and the mean number of collisions per lap in each condition are shown in Table 1. Taken together, these analyses demonstrate that subjects were able to perform the task successfully in all 10 conditions but reduced their speed when gates were not visible at least 1-1/2 segments in advance.

**Table 1.** Mean proportion of gates successfully completed and mean number of collisions per lap for each combination of configuration and lookahead distance.

	Configuration A					Configuration B				
	0.75	1	1.5	2	Full	0.75	1	1.5	2	Full
Proportion of gates completed	0.998	1.000	1.000	0.998	1.000	1.000	1.000	0.998	0.998	0.990
Collisions	0.813	0.750	0.688	1.188	0.938	0.813	1.000	0.813	1.000	1.432

### Steering behavior

**Figure 4.**
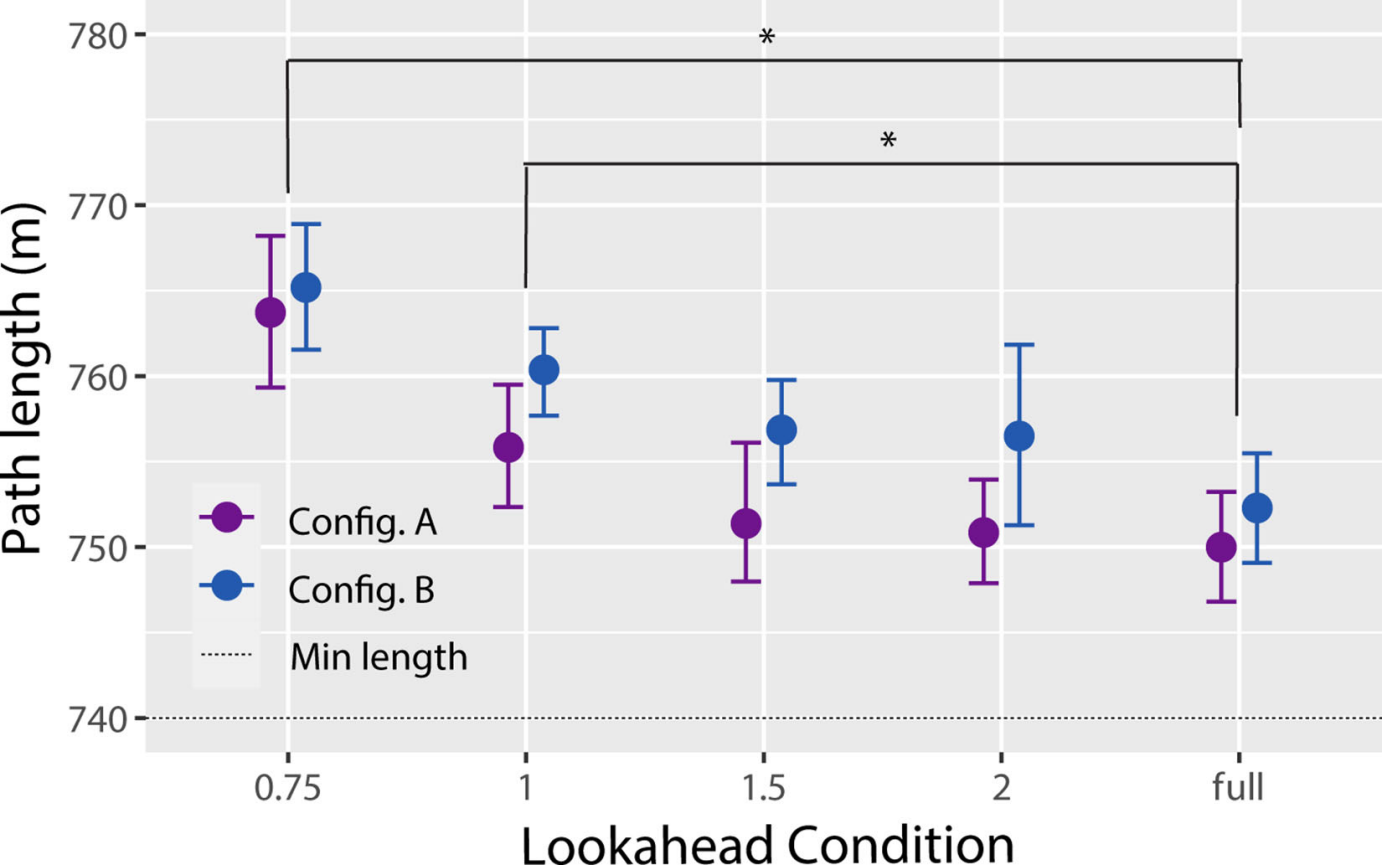
Path length as a function of lookahead distance for Configuration A (purple markers) and Configuration B (blue markers). Error bars are 95% confidence intervals with between-subjects variance removed. Asterisks indicate conditions that are significantly different (*p* < 0.05). The dotted line indicates the cumulative length of the linear distance between successive gates.

The previous set of analyses demonstrated that, when lookahead distance was shorter, subjects were still able to successfully steer through gates and avoid collisions but had to reduce speed to maintain performance. In this section, we consider how the manipulation of lookahead distance affected path length, steering smoothness, and the activity of the controllers that subjects used to adjust forward thrust and yaw rate. As shown in Figure 4, the total lengths of the trajectories that subjects followed tended to increase when lookahead distance was shorter, *F*(4, 60) = 8.20, *p* < 0.001, η^2^ = 0.16. Planned comparisons indicated that path length was significantly longer in the LD-0.75 and LD-1 conditions compared with the LD-Full condition. This suggests that when gates became visible more than one segment in advance, subjects were able to use information from those gates to follow more direct paths. Path length was also shorter in Configuration A compared with Configuration B, *F*(1, 15) = 25.00, *p* < 0.001, η^2^ = 0.06, which is not surprising given that a subset of gates in Configuration B was offset from the path. The lookahead distance × configuration interaction was not significant, *F*(4, 60) = 0.54, *p* = 0.71, η^2^ = 0.02.

**Figure 5.**
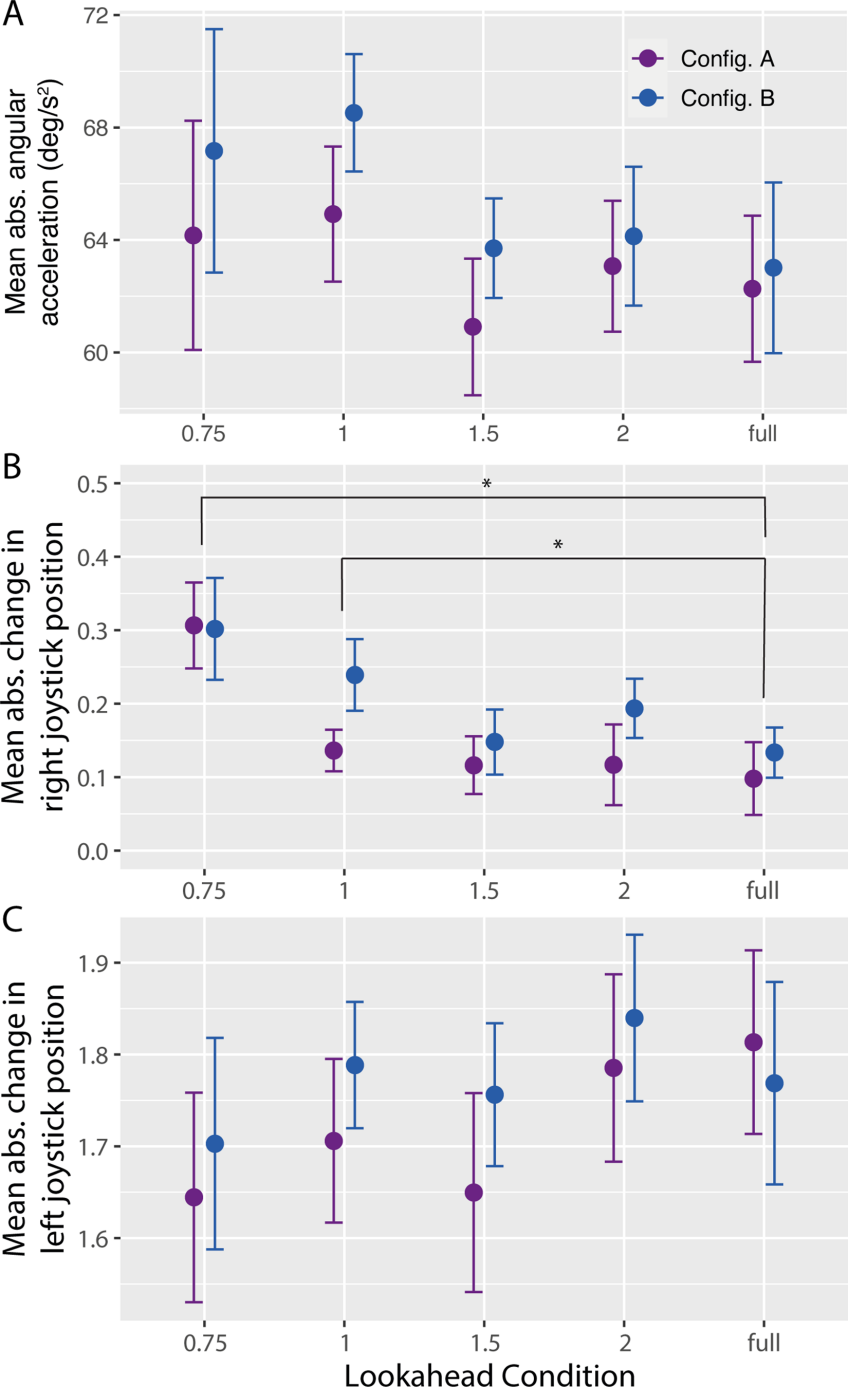
Mean absolute angular acceleration (**A**) and mean absolute change in right (**B**) and left (**C**) joystick position as a function of lookahead distance for Configuration A (purple markers) and Configuration B (blue markers). Joystick position was measured on a scale of −1 (full downward or leftward position) to +1 (full forward or rightward position), with zero corresponding to the neutral point. Error bars are 95% confidence intervals with between-subjects variance removed. Asterisks indicate conditions that are significantly different (p < 0.05).

Figure 5A shows mean absolute angular acceleration, which was used by Wilkie et al. (2008) as a measure of steering smoothness. The pattern was largely consistent with that in the path length analysis, with smoother paths found when lookahead distance was at least 1.5 segments and in Configuration A. Angular acceleration was higher, indicating more steering adjustments, when lookahead distance was shorter, *F*(4, 60) = 2.66, *p* < 0.05, η^2^ = 0.10. However, planned comparisons did not show a strong difference between each shorter lookahead condition and LD-Full. Angular acceleration was also higher in Configuration B than in Configuration A, which is consistent with the varied position and rotation of some gates in Configuration B, *F*(1, 15) = 8.50, *p* < 0.05, η^2^ = 0.04. The lookahead condition × orientation condition interaction was not significant, *F*(4, 60) = 0.59, *p* = 0.67, η^2^ = 0.01. The overall range of angular accelerations (60°/s^2^–70°/s^2^) was more than twice that in Wilkie et al. (2008) (25°/s^2^–30°/s^2^), which we attribute to differences in the task constraints, as subjects in the present study traveled at a higher speed (10–12 m/s vs. 8 m/s in Wilkie et al.) and the gates were closer together (∼23 m vs. 20–40 m), which means that subjects had to alter their trajectories more rapidly to steer through gates.

Activity of the right joystick, which controls forward and backward thrust, significantly varied across lookahead distance conditions, *F*(4, 60) = 22.44, *p* < 0.001, η^2^ = 0.41, with planned comparisons revealing significant differences (*p* < 0.05) between the LD-0.75 and LD-1 conditions and the LD-Full condition (see Figure 5B). Likewise, the effect of configuration was significant, *F*(1, 15) = 11.63, *p* < 0.005, η^2^ = 0.12, but the interaction was not, *F*(4, 60) = 1.39, *p* = 0.25, η^2^ = 0.03. Activity in the left joystick, which controlled the rotation (or yaw) rate of the drone, was greater overall than right joystick activity but was less strongly affected by lookahead distance, *F*(4, 60) = 2.56, *p* < 0.05, η^2^ = 0.09 (see Figure 5C). Neither the main effect of configuration, *F*(1, 15) = 2.67, *p* = 0.12, η^2^ = 0.02, nor the interaction, *F*(4, 60) = 0.86, *p* < 0.49, η^2^ = 0.02, was significant.

Taken together, these analyses provide some insight into how subjects modulated speed and steering across the different lookahead distance conditions. The increase in right joystick activity in the short LD conditions indicates that subjects did not simply fly at a slower speed when lookahead distance was shorter. Speed control was also more variable, with larger amplitude and more frequent changes to the joystick that controlled forward and backward thrust. Subjects also followed longer paths and exhibited higher rates of angular acceleration in the shorter LD conditions. Taken together, these analyses demonstrate that, when information from beyond the most immediate gate is available, humans can use it to maintain a faster and more consistent speed, move along shorter paths, and steer more smoothly.

### Prospective steering analyses

**Figure 6.**
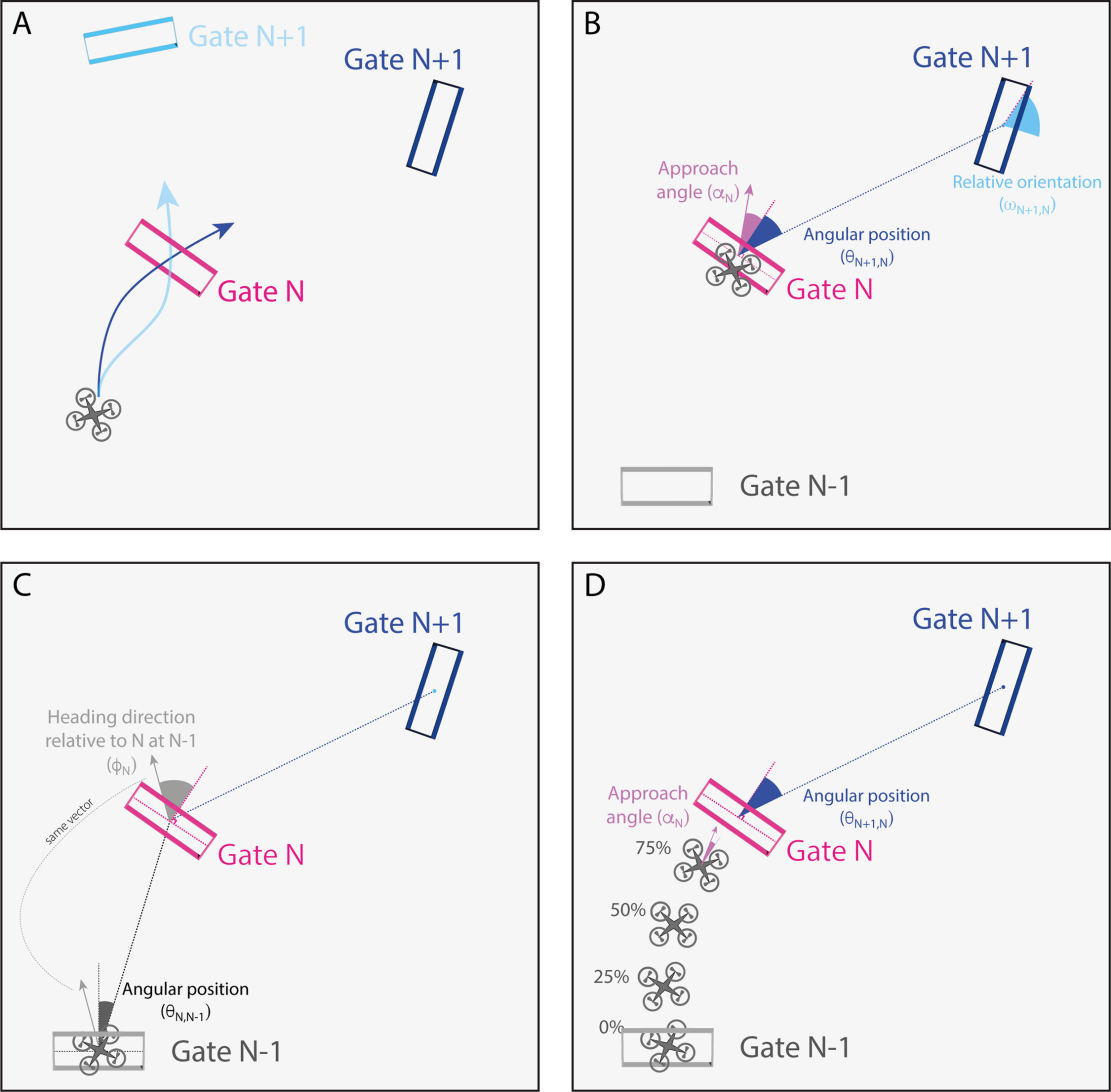
(**A**) Top–down view illustrating how the trajectory to the most immediate gate (Gate*_N_*) may depend on position of subsequent gate (Gate*_N_*_+1_). (**B**, **C**) Predictor variables (angular position and relative orientation), outcome variable (approach angle), and covariates (heading direction relative to *N* at *N*–1, angular position of *N* relative to *N*–1) used in the linear model. (**D**) Illustration of outcome variable (approach angle) measured at different positions leading up to Hoop *N*. Figure adapted from Powell et al. (2024).

If subjects adapted how they approached the immediate gate (Gate*_N_*) in a way that depended on the position of the next gate (Gate*_N_*_+1_), the quadcopter approach angle to Gate*_N_* should vary in a way that depends on the angular position of Gate*_N_*_+1_ relative to Gate*_N_* (see Figure 6A). We defined approach angle (⍺*_N_*) as the instantaneous heading of the drone at the instant the drone passed through Gate*_N_* relative to the normal vector of Gate*_N_* (see Figure 6B). The angular position of Gate*_N_*_+1_ (θ*_N_*_+1,_*_N_*) was defined as the angle between the vector connecting the centers of Gate*_N_* and Gate*_N_*_+1_ and the normal vector of Gate*_N_* (see Figure 6B). If subjects anticipated Gate*_N_*_+1_ during their approach to Gate*_N_*, the angular position of Gate*_N_*_+1_ should be a significant predictor of approach angle to Gate*_N_*.

To test this prediction, we fit a linear model to the data using angular position of Gate*_N_*_+1_ as the predictor (θ*_N_*_+1,_*_N_*) and approach angle to Gate*_N_* as the outcome variable (⍺*_N_*). The approach angle to Gate*_N_* may also be affected by the state of the drone at the instant that it passes through Gate*_N_*_–__1_ (i.e., the initial conditions for that segment). As such, we included two covariates to account for possible variance related to the initial state of the drone for the segment between Gate*_N_*_–__1_ and Gate*_N_*: (1) the heading direction of the drone relative to the normal vector of Gate*_N_* at Gate*_N_*_–__1_ (ɸ*_N_*) and (2) the angular position of Gate*_N_* relative to Gate*_N_*_–__1_ (θ*_N_*_,_*_N_*_–__1_) (see Figure 6C). Equation 2 describes the full model that was fit to the data:

(2) $$\begin{array}{c} \alpha_{N}∼\phi_{N}+\theta_{N,N-1}+\theta_{N+1,N}\; \end{array}$$

**Figure 7.**
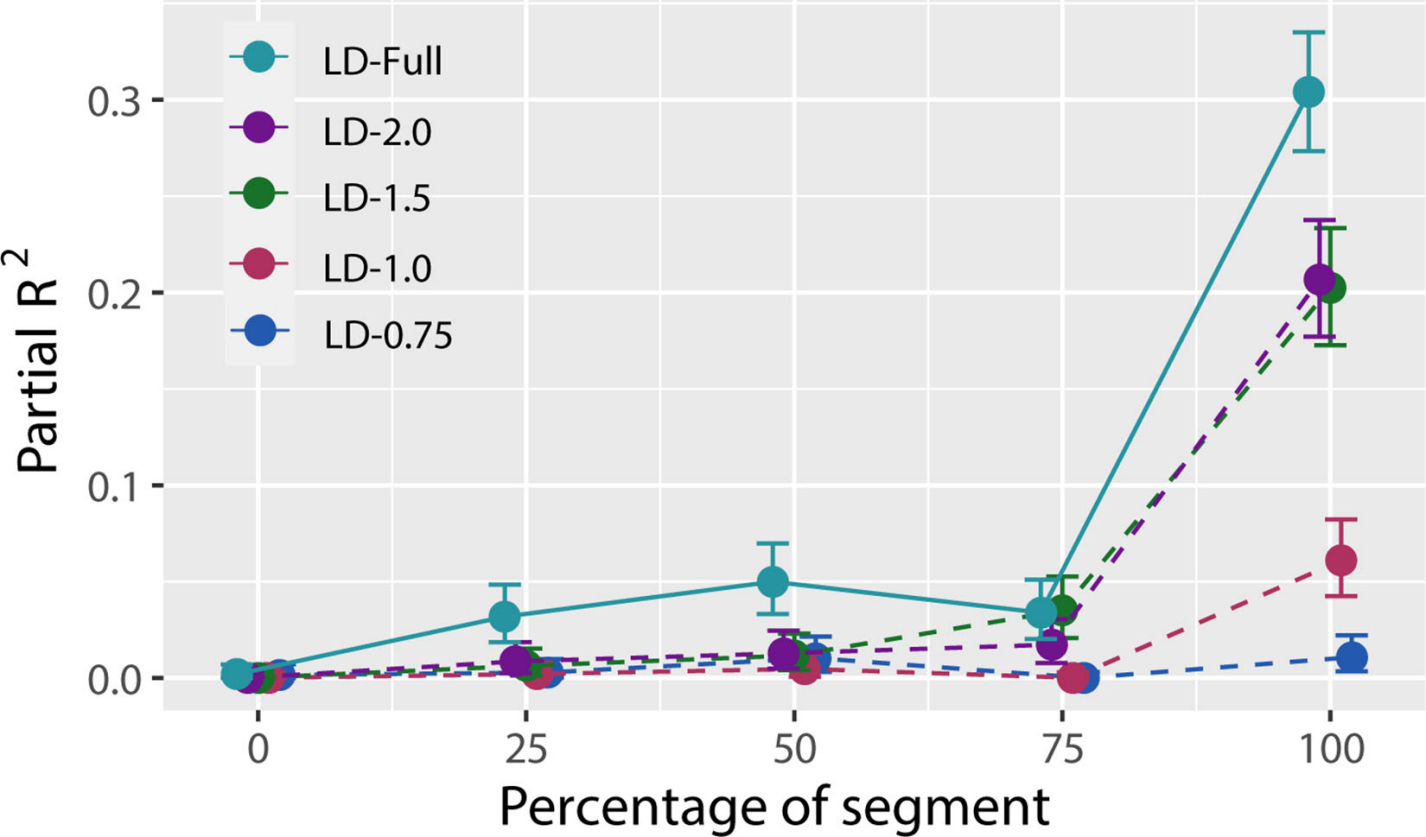
Partial *R*^2^ (proportion of residual variance explained by the angular position of Gate*_N_*_+1_, which is θ*_N_*_+1,_*_N_* in Equation 2) as a function of the percentage of segment for different LD conditions. Solid lines indicate the LD-Full condition, and dashed lines are used for conditions with manipulated lookahead distances. Error bars correspond to 95% confidence intervals.

In the Full lookahead condition, the angular position of Gate*_N_*_+1_ was indeed a significant predictor of approach angle to Gate*_N_*, accounting for 30.4% (95% confidence interval [CI], 27.3–33.5) of the variance that was unexplained by the covariates. This indicates that, at some point before the drone reached Gate*_N_*, subjects had already started to anticipate having to steer through Gate*_N_*_+1_. To better understand how far in advance subjects started to use information about Gate*_N_*_+1_, we repeated the analysis with approach angle measured at four earlier points in the segment between Gate*_N_*_–__1_ and Gate*_N_*: when the drone was 75%, 50%, 25%, and 0% of the way to reaching Gate*_N_* (see Figure 6D). Intuitively, the proportion of variance explained should decrease as ⍺*_N_* is measured at earlier points in time, which is borne out in the results (see Figure 7).

A substantial proportion of variance in drone approach angle at Gate*_N_* (100% of the segment) was also explained by the relative position of Gate*_N_*_+1_ in the LD-2.0 (20.7%) and LD-1.5 (20.2%) conditions, but not in the LD-1.0 (6.0%) or LD-0.75 (1.0%) conditions (see Figure 7). These findings are consistent with the analyses of performance measures and steering behavior and provide further evidence that subjects used information from the *N*+1 gate when it was available.

Next, we repeated the analysis with the main predictor variable changed to the orientation of Gate*_N_*_+1_ relative to Gate*_N_* (ω*_N_*_+1,_*_N_*) (Figure 6B):

(3) $$\begin{array}{c} \alpha_{N}∼\phi_{N}+\theta_{N,N-1}+\omega_{N+1,N}\; \end{array}$$

None of the partial *R*^2^ values exceeded 0.008 in any of the lookahead distance conditions, even at 100% of the segment. Thus, although subjects adapted their trajectory based on the angular position of Gate*_N_*_+1_, we found no evidence that they took the orientation of the *N*+1 gate into account.

### Analyses of gaze behavior

The analyses in this section address the question of whether subjects made gaze shifts to Gate*_N_*_+1_ and, if so, how such eye movements may be related to the prospective control of steering. We focus on data from the LD-Full condition due to concerns that the manipulation of gate visibility in the other LD conditions disrupted normal gaze behavior.

**Figure 8.**
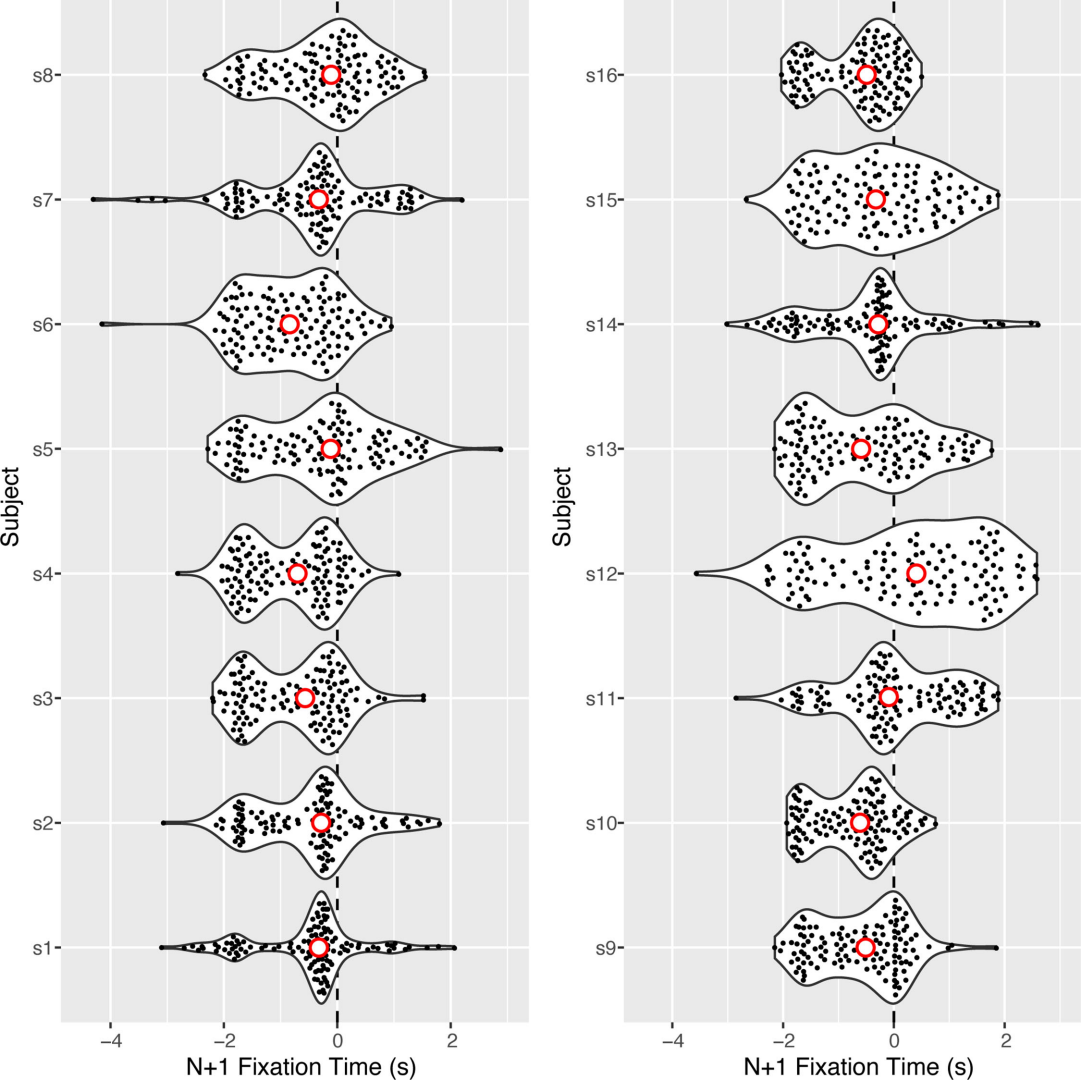
Violin plots of the distribution of *N*+1 fixation times for each subject. Negative and positive values correspond to fixations of Gate*_N_*_+1_ prior to and after reaching Gate*_N_*, respectively. The open red circles indicate the median *N*+1 fixation times for each subject.

For each pair of gates, we measured the amount of time before passing through Gate*_N_* that subjects initially fixated Gate*_N_*_+1_. Figure 8 shows the distribution of these Gate*_N_*_+1_ fixation (henceforth, *N*+1 fixation) times for each individual subject. Negative and positive values correspond to initial fixations of Gate*_N_*_+1_ before and after reaching Gate*_N_*, respectively. The median *N*+1 fixation times were negative for all but one of the 16 subjects, and the mean percentage of negative *N*+1 fixation times across subjects was 70.1% (95% CI, 55.8–84.4), indicating that subjects initially fixated Gate*_N_*_+1_ before reaching Gate*_N_* more often than not.

Figure 8 also reveals that *N*+1 fixation times were bimodally distributed and quite variable, ranging between approximately −2 and +0.5 seconds for most subjects. This is partly because some gates were positioned along straightaways and plainly visible well in advance but others were located around sharp curves and visually occluded by foliage. Indeed, the distribution of *N*+1 fixation times was notably shifted in the negative direction for Gates 11 to 14 (marked by a negative sign in Figure 1B), which were positioned on a straight section of the path. Likewise, *N*+1 fixation times were shifted in the positive direction for gates that were positioned around a tree-line bend (e.g., Gates 7, 20, 22, and 26, indicated by plus signs in Figure 1B).

**Figure 9.**
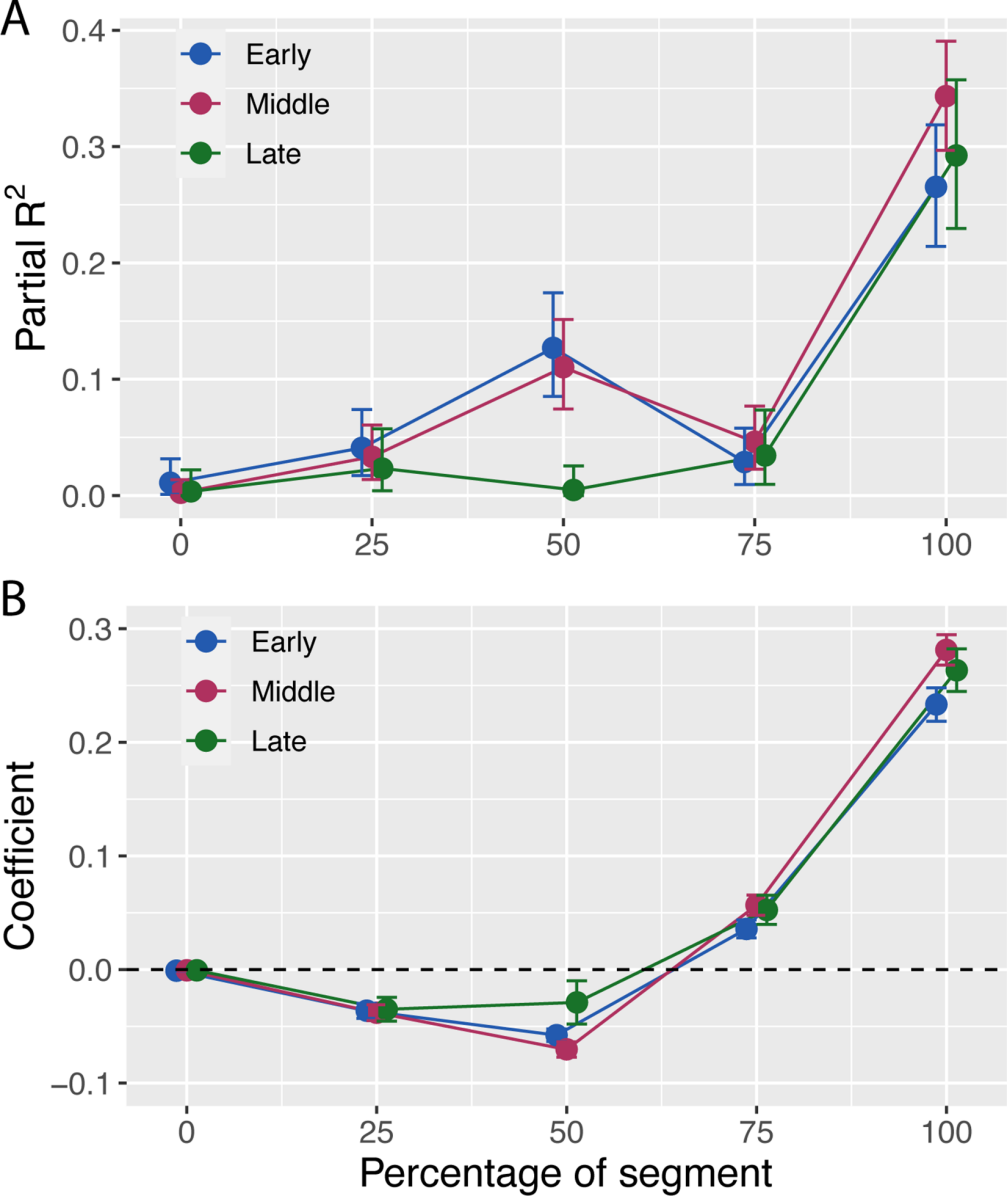
(**A**) Partial *R*^2^ (proportion of residual variance explained by the angular position of Gate*_N_*_+1_, which is θ_*N*+1,*N*_ in Equation 2) as a function of the percentage of segment for different *N*+1 fixation time categories. (**B**) Coefficients for θ*_N_*_+1,_*_N_* as a function of the percentage of segment for different *N*+1 fixation time categories. Error bars correspond to 95% confidence intervals.

To determine whether *N*+1 fixations were related to prospective steering adjustments, we separated segments into bins based on whether the *N*+1 fixation time was early (less than −1 second; 696 values), middle (between −1 and 0 seconds; 852 values), or late (greater than 0 seconds; 500 values). We then re-ran the analysis in the previous section, fitting Equation 2 to the data from segments with early, middle, and late *N*+1 fixation. Figure 9A shows the proportion of variance (partial *R*^2^) in approach angle to Gate*_N_* (⍺*_N_*) that was explained by the angular position of Gate*_N_*_+1_ (θ*_N_*_+1,_*_N_*) broken down by percentage of segment and Gate*_N_*_+1_ fixation time. When the approach angle to Gate*_N_* was measured at the end of the segment (100%), the proportion of variance explained by Gate*_N_*_+1_ position was similar (∼30%) for all three categories of *N*+1 fixation time. In addition, the coefficient for θ*_N_*_+1,_*_N_* was positive for all three *N*+1 fixation time categories (Figure 9B), indicating that, when Gate*_N_*_+1_ was to the right, subjects tended to be heading to the right as they passed through Gate*_N_* (and vice versa). Thus, even when subjects did not initially fixate Gate*_N_*_+1_ until after passing through Gate*_N_*, they still made prospective steering adjustments. This suggests that such adjustments can precede *N*+1 fixation.

Although there were no differences across *N*+1 fixation time categories at 100%, partial *R*^2^ was higher for early and middle *N*+1 fixations compared with late *N*+1 fixations at 50% of the segment. Furthermore, the coefficients were negative, indicating that, on trials with early and middle *N*+1 fixations, approach angle relative to Gate*_N_* (measured at the midpoint of the segment) was negatively correlated with the angular position of Gate*_N_*_+1_. We offer the following as a tentative interpretation of this result: When Gate*_N_*_+1_ was visible far enough in advance, subjects were more likely to make earlier *N*+1 fixations, as well as prospective steering adjustments. The latter could account for the negative correlation, as such adjustments would involve initially steering in the direction opposite from Gate*_N_*_+1_ (to allow for a smoother trajectory through Gate*_N_* to Gate*_N_*_+1_). Furthermore, at some point between 50% of the segment (when approach angle was negatively correlated with Gate*_N_*_+1_ angle) and 100% of the segment (when approach angle was positively correlated with Gate*_N_*_+1_ angle), subjects would have to be moving in a direction that was neither the same as nor different from the Gate*_N_*_+1_ angle. This could account for the drop in partial *R*^2^ at 75% of the segment in the early and middle *N*+1 fixation time categories.

Taken together, these findings provide evidence that *N*+1 fixations are related to prospective steering adjustments. Specifically, when subjects made *N*+1 fixations before reaching Gate*_N_*, they also tended to make earlier prospective steering adjustments that depended on the angular position of Gate*_N_*_+1_. In the Discussion, we consider the possible nature of this relationship.

## Discussion

We begin the Discussion with the main question that motivated this study: When humans perform tasks that involve steering through multiple waypoints, do they use information from beyond the most immediate waypoint to improve performance? The evidence strongly indicates that they do. In the absence of manipulations of visual lookahead distance (i.e., when all gates were rendered in the scene from the onset), subjects successfully flew through a high percentage (*M* = 99.80%) of gates, rarely collided with obstacles (*M* = 0.94 per lap), and maintained an average flight speed of 10.95 m/s, which is 87.56% of maximum speed. They also followed paths that were only 2.28% longer than the cumulative linear distance between pairs of gates, and they adapted how they approached the most immediate gate in a way that depended on the angular position of the subsequent gate. However, when visual lookahead distance was decreased, such that gates did not appear until they fell within 1-1/2 segments of the quadcopter, subjects reduced their speed and increased activity in the right joystick, which controlled forward–backward thrust. They also followed longer paths and were less able to adjust their approach angle to the upcoming gate in a way that depended on the subsequent gate.

Taken together, the findings provide compelling evidence that humans are capable of using information from beyond the most immediate waypoint to move at a faster, more consistent speed while traveling along shorter, more linear trajectories. The results are consistent with those of Powell et al. (2024), who also found evidence of anticipation in a similar task; namely, the approach angle to the upcoming hoop was systematically related to the angular position of the subsequent hoop. However, subjects in that experiment had extensive FPV drone piloting experience. The present study demonstrates that anticipation of future waypoints is possible even in actors who lack extensive experience with high-speed steering and obstacle avoidance in cluttered environments. In addition, Powell et al. (2024) did not manipulate lookahead distance.

The findings of the present study complement those of Wilkie et al. (2008), who found only weak and inconsistent effects on steering smoothness when waypoints were made visible farther in advance. We attribute this to differences in the task difficulty of the two studies. In Wilkie et al., subjects traveled at a fixed, slower speed; gates were spaced farther apart along the longitudinal axis and oriented in the same direction; and there were no obstacles in the scene. As such, even if subjects glanced ahead to the *N*+1 gate, the effects of information about that gate on steering behavior may have been negligible.

### Gaze behavior

As in previous studies (Wilkie et al., 2008), subjects in the present study often shifted gaze to Gate*_N_*_+1_ before passing through Gate*_N_*. *N*+1 fixation times were quite variable, partly because some gates were visible well in advance but others were positioned around sharp, tree-lined curves. Nevertheless, when subjects did shift gaze to Gate*_N_*_+1_ before reaching Gate*_N_*, they also tended to make earlier steering adjustments that depended on the position of Gate*_N_*_+1_. This does not necessarily mean that *N*+1 fixations caused prospective steering adjustments. In fact, the evidence suggests that subjects sometimes initiated prospective steering adjustments prior to making an *N*+1 fixation. For example, the approach angle at 100% was positively related to the angular position of Gate*_N_*_+1_ even when fixations to Gate*_N_*_+1_ were not made until after passing through Gate*_N_* (“late” in Figure 9). This suggests that initial fixations to Gate*_N_*_+1_ sometimes followed rather than preceded prospective steering adjustments. It is also possible that both early *N*+1 fixations and prospective steering adjustments were determined by another variable, such as how far in advance the *N*+1 gate became visible. Further research is needed to tease apart these possibilities.

### Manipulation of gate configuration

We also hypothesized that actors may need information from farther ahead when the waypoints are distributed in a less consistent pattern; however, there was a lack of evidence to support this hypothesis. If it is correct, we should have observed differences in performance relative to the LD-Full condition at longer lookahead distances in Configuration B compared with Configuration A. This would have manifested in a significant interaction between lookahead distance and configuration. Although there was a general trend in the analyses of mean speed (Figure 3) and path length (Figure 4) that was consistent with this prediction, the lookahead distance × configuration interaction was not statistically significant for either measure. It could be that manipulation of gate configuration was not strong enough; that is, the difference in consistency of gate positions and orientations between the configurations was too small to have a detectable effect on the importance of information about the *N*+1 gate. Alternatively, it is possible that the role of information about the *N*+1 gate is unaffected by the spatial layout of waypoints.

### Occurrent information or knowledge of course layout

Up to this point, we have assumed that the subjects’ ability to anticipate Gate*_N_*_+1_ was based on currently available information. In principle, however, subjects also could have relied on knowledge of the spatial layout of the course that was acquired while performing the experiment. After all, except for the nine gates whose position and orientation differed between the two configurations, the shape of the loop and the positions of the gates were the same for each of the 20 trials. However, if subjects relied on prior knowledge of course layout rather than currently available information, one would expect to find evidence of anticipation regardless of when gates became visible. This was not the case. As shown in Figure 7, the proportion of variance in approach angle that was accounted for by the position of the *N*+1 gate in the LD-1.0 and LD-0.75 conditions was negligible. Evidence of anticipation was only found when information about the *N*+1 gate was available.

### Control strategies

The findings of the present study contribute to the base of knowledge that is needed to begin to formulate possible prospective control strategies for steering through multiple waypoints. For example, the fact that approach angle to Gate*_N_* was systematically related to the angular position of Gate*_N_*_+1_ rules out any strategy that focuses entirely on the most immediate waypoint. One possible strategy that relies on information from the two upcoming waypoints is based on the behavioral dynamics model (Fajen & Warren, 2003), which treats goals and obstacles as attractors and repellers of heading, respectively. Zhao and Warren (2011) proposed a variation of the behavioral dynamics, with the two upcoming goals acting as attractors of heading. The relative influence of both goals is captured by a weight, which varies depending on the distance of the agent to the two nearest goals. More recently, Tuhkanen, Pekkanen, Mole, Wilkie, and Lappi (2023) proposed an alternative strategy according to which the fixated waypoint acts as an attractor of heading (see also Wilkie et al., 2008). Steering at any instant is guided by a single waypoint, but that waypoint can switch with changes in gaze. Thus, actors could anticipate future waypoints by strategically shifting gaze between them. A third possibility is that actors rely on information about the constant radius path that passes through the two upcoming waypoints and adjust steering to null the difference between the current path and the optically specified path (Fajen & Jansen, 2023). The findings of the present study clearly rule out any strategy that is based solely on the most immediate waypoint, but further research is needed to tease apart the predictions of the remaining models.

There are other aspects of human behavior revealed in the present study that could inform the development of possible control strategies for steering through multiple waypoints: (1) at least under the conditions used in the present study, 1-1/2 segments was the necessary and sufficient lookahead distance to reach the level of performance in the full vision condition; (2) the approach trajectory to waypoint *N* was affected by the position but not the orientation of waypoint *N*+1; and (3) subjects adapted to the decrease in lookahead distance by decreasing their speed. These aspects of human behavior can serve as useful benchmarks for evaluating potential models of steering through multiple waypoints.

## Supplementary Material

Supplement 1

Supplement 2

Supplement 3
